# Homogeneous Selection and Dispersal Limitation Drive Phyllosphere Fungal Community Assembly in Constructed Wetland Ecosystems

**DOI:** 10.3390/biology14101378

**Published:** 2025-10-09

**Authors:** Nan Deng, Yuxin Tian, Qingan Song, Yandong Niu, Fengfeng Ma

**Affiliations:** 1 Hunan Academy of Forestry, No. 658 Shaoshan Road, Changsha 410004, China; dengnan@hnlky.cn (N.D.);; 2 Hunan Cili Forest Ecosystem State Research Station, Changsha 410004, China; 3 Dongting Lake National Positioning Observation and Research Station of Wetland Ecosystem of Hunan Province, Yueyang 414000, China; 4 International Technological Cooperation Base for Ecosystem Management and Sustainable Utilization of Water Resources in Dongting Lake Basin, Changsha 410004, China

**Keywords:** constructed wetlands, phyllosphere fungi, community assembly, high-throughput sequencing, homogeneous selection

## Abstract

Constructed wetlands (CWs) are widely used for ecological restoration, but their microbial assembly mechanisms and ecological functions remain poorly understood, limiting the optimization of restoration strategies. In this study, we investigated foliar fungal communities associated with dominant plant types in a CW converted from farmland by employing high-throughput sequencing. We analyzed community composition, diversity, assembly processes, and co-occurrence network structure to identify potential keystone taxa. Our results showed that plant type significantly influenced fungal community diversity and composition (α < 0.05). Assembly processes were jointly driven by heterogeneous selection (36.48%), dispersal-related processes (35.49%), and drift (24.70%), indicating comparable contributions of deterministic and stochastic processes. Co-occurrence network analysis revealed a modular structure and identified several hub taxa based on topological indices, suggesting their potential importance for network stability. This study provides an integrated perspective that links community diversity, assembly mechanisms, and network structure, providing insights for improving CW vegetation management and microbial regulation strategies. Future studies should explore and validate the functional roles of hub taxa and test the generalizability of these patterns across multiple regions and seasons.

## Simple Summary

Wetlands are important ecosystems that help clean water, store carbon, reduce flooding, and provide habitat for wildlife. Many natural wetlands have been lost due to farming and development, and one solution is to create new “constructed wetlands”. In these human-made systems, plants and the tiny organisms living on them play a major role in keeping the ecosystem healthy. Among these organisms, fungi that live on leaves are especially important because they can affect plant growth, disease resistance, and overall ecosystem stability. In this study, we looked at fungi living on the leaves of different plant species in a constructed wetland that was converted from farmland. We found that the type of plant strongly influenced the kinds of fungi present. Both environmental conditions and chance events shaped how fungal communities formed, with neither completely dominating. We also identified a few key fungal species that appear to play central roles in the community and may help maintain stability. Our findings show that choosing certain plants can influence fungal communities, and managing these relationships may help improve the design and success of constructed wetlands for environmental restoration.

## 1. Introduction

Wetlands are among the most productive and ecologically valuable ecosystems on earth, performing critical ecosystem services including wastewater treatment, flood mitigation, wildlife habitat provision, and carbon sequestration [1,2]. These ecosystems are characterized by extensive anaerobic soil conditions and serve as crucial components of global biogeochemical cycles, particularly for carbon and nitrogen cycling [3]. However, anthropogenic activities have severely threatened wetland ecosystems, with more than half of the earth’s wetlands altered or degraded over the past 150 years [4,5], making them one of the most threatened ecosystems globally. To address extensive wetland loss, constructed wetlands (CWs) have emerged as a promising nature-based solution for ecosystem restoration and wastewater treatment [6]. These engineered systems replicate natural wetland functions by utilizing physical, chemical, and biological processes, leading to their widespread global adoption as cost-effective restoration technologies [7]. Among restoration approaches, farmland-to-wetland conversion has become increasingly common due to agricultural intensification impacts on water quality and biodiversity [8]. Previous studies have demonstrated that such conversions can effectively restore ecosystem functions, though microbial community responses remain poorly characterized [9,10].

The efficacy of constructed wetlands fundamentally depends on complex interactions between vegetation and associated microorganisms [11,12]. Plants facilitate microbial colonization through attachment to root surfaces and biofilm formation, creating diverse microenvironments that support varied ecological processes [13]. Different plant species exhibit unique root architectures and release distinct exudates, thereby supporting diverse microbial communities with varying metabolic capacities [14]. While both bacterial and fungal communities contribute to wetland functioning, fungi warrant particular attention in restoration contexts for several reasons. First, fungi exhibit greater sensitivity to environmental perturbations compared to bacteria, making them valuable early indicators of ecosystem recovery [15]. Second, fungal networks facilitate plant establishment and stress tolerance through mycorrhizal associations, which are particularly crucial during restoration phases [16]. Third, phyllosphere fungi, compared to rhizosphere communities, provide direct insights into above-ground plant–microbe interactions that influence plant health and community dynamics [17,18]. Fungal community diversity serves as a valuable indicator for assessing soil nutrient status, environmental quality, and overall ecosystem health and stability [19,20]. Studies have shown that wetland degradation leads to significant declines in fungal community diversity, with composition and species diversity varying considerably across different degradation stages [21,22,23].

Despite growing recognition of plant–microbe interactions in wetland ecosystems, critical knowledge gaps persist regarding phyllosphere fungal communities in restored wetlands. Previous research has primarily focused on soil microbial communities (e.g., [24]), with limited attention to phyllosphere assemblages despite their direct influence on plant health and ecosystem stability [25]. Existing studies of phyllosphere fungi have been largely focused on natural wetlands or agricultural systems [26,27], leaving the assembly processes of different vegetation types in restored wetlands poorly understood. This knowledge gap hinders optimization of restoration strategies through targeted vegetation selection. Furthermore, the mechanisms driving fungal community assembly in constructed wetlands remain inadequately characterized [28,29]. Understanding these assembly mechanisms is essential because they determine ecosystem stability, resilience, and functional recovery following restoration interventions [30,31]. This study addresses these knowledge gaps by investigating a constructed wetland system established through farmland-to-wetland conversion, which features multiple vegetation types and management patterns, providing an ideal natural laboratory for examining plant–fungal interactions. This study aims to characterize diversity patterns of phyllosphere fungal communities associated with different vegetation types in constructed wetland systems. The research will elucidate the assembly processes governing these phyllosphere fungal communities while identifying their key environmental drivers and determining the critical environmental factors that influence fungal community structure and function in restored wetland ecosystems. This research will provide crucial insights into the ecological dynamics governing microbial communities in restored wetlands, thereby informing evidence-based restoration strategies that optimize ecosystem function recovery.

## 2. Materials and Methods

### 2.1. Study Area and Sample Collection

**Figure 1:**
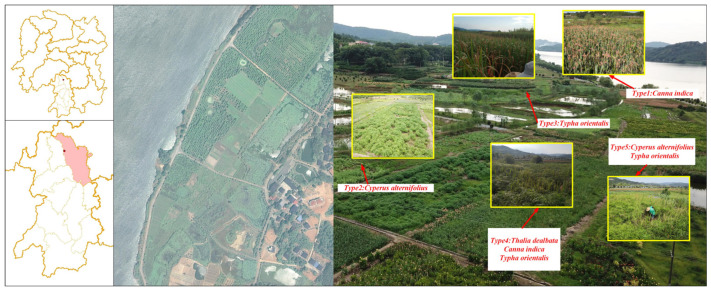
Layout of five experimental plots.

Our study was conducted in the demonstration area for returning farmland to wetland in Xiangjia Village, Yongzhou City, Hunan Province, China. The study area consists of river wetlands in the middle reaches of the Xiangjiang River Basin (Geographic coordinates: 111°45′58.0824″ E, 26°34′36.6204″ N). The region experiences a subtropical monsoon humid climate, with an annual average temperature of 18.2 °C and annual average precipitation of 1275.7 mm. The climate is characterized by rainy summers and dry autumn–winter seasons. Prior to wetland conversion, this area consisted of densely populated agricultural land that relied on river irrigation for farming activities. To address regional agricultural non-point source pollution, the Forestry Department of Hunan Province initiated a demonstration project in 2019, implementing wetland restoration measures across a three-hectare area. Various constructed wetlands were established as part of this restoration effort, with wetland plant cultivation proving particularly effective for ecological recovery. Five constructed wetland plots with different plant compositions were selected (Figure 1 and Table 1): *Cyperus alternifolius* L., *Canna indica* L., *Typha orientalis* Presl, *Thalia dealbata Fraser* + *Typha orientalis* + *Canna indica*, and *Typha orientalis* + *Cyperus alternifolius*. Each plot covered an area of 800 m^2^ and was separated from adjacent plots by earthen banks (40–50 cm height).

At each plot, three small plots (1 m^2^) separated by intervals of more than 10 m were selected. The growth parameters of plants, including height and coverage, were measured, and then all plants within each plot were harvested to collect samples weighing about 500 g. This sampling strategy provided three independent biological replicates for each of the five plant types, resulting in a total of 15 samples for subsequent analysis. To obtain phyllosphere samples, at least four fully expanded healthy leaves were sampled from each of the four cardinal directions (east, north, west, and south) within each plot. Healthy leaves were defined as those that were intact without damage, free from obvious disease spots, insect damage, and with normal leaf coloration. The collected leaves were preserved at 4 °C and processed within 3 h of collection. The leaves were promptly immersed in fixative (1×PBS Buffer, Coolaber, Beijing, China) at 4 °C, then shaken on the shaking table for 30 min (200 rpm). The oscillating solution was then filtered through a 0.22 µm membrane filter.

### 2.2. High-Throughput Sequencing and Bioinformatics Analyses

Microbial DNA was extracted from samples using the FastDNA Spin kit (MP Biomedicals, Santa Ana, CA, USA) following the manufacturer’s instructions. The fungal ITS1 region was then amplified. High-throughput sequencing was conducted on an Illumina MiSeq platform (Illumina, Inc., San Diego, CA, USA). Raw sequence data were processed and analyzed with QIIME2 (version 2019), following the workflow outlined at https://qiime2.org [32]. OTUs were identified using the UPARSE pipeline (v7.0.1).

### 2.3. Physicochemical Properties Analyses

The harvested plants were separated into above-ground and below-ground parts for separate measurements. The total biomass (kg/m^2^) was calculated. Soil samples were collected from beneath each plant. Total nitrogen, phosphorus, potassium, and soil organic carbon (SOC) were measured. Total nitrogen content was using the Kjeldahl digestion method, which involved digestion with concentrated sulfuric acid, followed by steam distillation and subsequent titration [33,34]. Total phosphorus was quantified using sulfuric acid–perchloric acid digestion, followed by the Mo-Sb colorimetric method [35]. Total potassium content was quantified by NaOH fusion-flame photometry [36]. Soil organic carbon (SOC) content was quantified using the potassium dichromate oxidation-titration method [37].

### 2.4. Statistical Analyses

Diversity calculation: Alpha diversity indices including observed species, good’s coverage, Chao1, phylogenetic diversity (PD whole tree) whole tree, Simpson, and Shannon indices were calculated using the ‘vegan’ package in R (version 4.3). These indices provide complementary measures of community richness, evenness, and phylogenetic structure. Chao1 estimates species richness accounting for rare species, while Simpson and Shannon indices capture both evenness and richness aspects of diversity. Phylogenetic diversity (PD) whole tree quantifies the total phylogenetic branch length represented in each community, providing insights into evolutionary diversity.

To investigate Beta diversity decomposition, Bray–Curtis dissimilarity matrices were subsequently decomposed into three additive components: replacement (turnover), richness difference (nestedness), and total dissimilarity [38]. The analyses were performed using the R (version 4.3) package of ‘picante’ (version 1.8.2) [39], ‘vegan’ [40], and ‘ape’ (version 5.7-1) [41]. The matrices of β-diversity were visualized using ternary plots, where each point corresponds to the decomposed values of replacement, richness difference, and similarity in pairwise community comparisons.

Ternary plots were constructed using the ‘ggtern’ package (version 3.4.2) [42] in R to visualize compositional data with three components that sum to 100%. In ternary plots, each point’s position is determined by the relative proportions of three variables, with the three vertices representing 100% contribution of each respective component. Points closer to a vertex indicate higher relative contribution of that component. Ternary plots were applied to represent both phylum-level composition differences among plant types and Beta diversity decomposition components.

Multiple comparative analysis: Multiple comparative analysis was performed to detect significant differences among different plant types at the 95% family-wise confidence level. Prior to conducting multiple comparisons, the normality assumption was verified using the Shapiro–Wilk test for each index, with all *p*-values exceeding 0.05, indicating that the data met the normality assumption. The Tukey HSD [43] test was selected to control for Type I error inflation when performing multiple pairwise comparisons between the five wetland types. This post hoc test maintains the experiment-wise error rate at α = 0.05 while providing simultaneous confidence intervals for all pairwise differences. The analysis was performed using the ‘multcomp’ package in R, with results visualized as confidence interval plots. In these plots, each comparison pair (e.g., type2-type1, type3-type1, etc.) is represented by a point estimate of the mean difference and horizontal error bars indicating the 95% confidence interval. Significant differences are identified when the confidence interval does not cross the vertical reference line at zero, indicating that the null hypothesis of no difference can be rejected.

Community assembly mechanism analysis (iCAMP): To reveal phyllosphere fungal community assembly processes, the iCAMP model (infer Community Assembly Mechanisms by Phylogenetic-bin-based null model analysis) was utilized using the ‘iCAMP’ package (version 1.8.2) [44] in R. The analysis began with phylogenetic binning, where the fungal community was divided into 21 taxonomic bins based on phylogenetic relationships, with this binning approach grouping phylogenetically related taxa that likely share similar ecological traits and environmental responses. Subsequently, null model analysis was conducted to examine five assembly processes based on bin-based approach, including deterministic processes of homogeneous selection (HoS) and heterogeneous selection (HeS), as well as stochastic processes encompassing homogenizing dispersal (HD), dispersal limitation (DL), and drift and other processes (DR), with the relative contribution of five process quantified by comparing observed patterns to null expectations generated through randomization. Finally, environmental driver identification was performed for bins associated with non-neutral processes (bins with HoS values exceeding 0.16) through Mantel tests between the corrected BetaMNTD dissimilarities for each bin and environmental variables, with partial Mantel correlations computed to control for confounding environmental factors. To assess statistical uncertainty, bootstrap resampling was performed with 100 iterations by resampling the community matrix with replacement and running the complete iCAMP analysis on each bootstrap dataset.

Co-occurrence network analysis: To examine the co-occurrence patterns and potential ecological interactions among fungal taxa in the phyllosphere, a co-occurrence network analysis was performed. Pairwise Spearman correlation coefficients were calculated among OTUs. Correlations with |r| > 0.6 and *p* < 0.01, after FDR correction were retained for network construction. The network’s modular structure was delineated through the application of the fast-greedy modularity optimization algorithm. Topological roles of each OTUs within the network were assessed using two metrics: participation coefficient (Pi) and within-module degree (Zi). Based on the framework proposed by Guimerà and Amaral [44], OTUs were categorized into four roles:

Peripheral nodes (Pi ≤ 0.62, Zi ≤ 2.5): weakly connected outside their module;

Connectors (Pi > 0.62, Zi ≤ 2.5): important for linking modules;

Module hubs (Pi ≤ 0.62, Zi > 2.5): central within their module;

Network hubs (Pi > 0.62, Zi > 2.5): highly connected across modules.

The resulting correlation matrix was visualized using the R package igraph [45]. In addition, role classification and Zi-Pi distribution plotting were performed within the same analytical framework using igraph.

## 3. Results

### 3.1. Alpha Diversity Index of Phyllosphere Fungi

**Figure 2:**
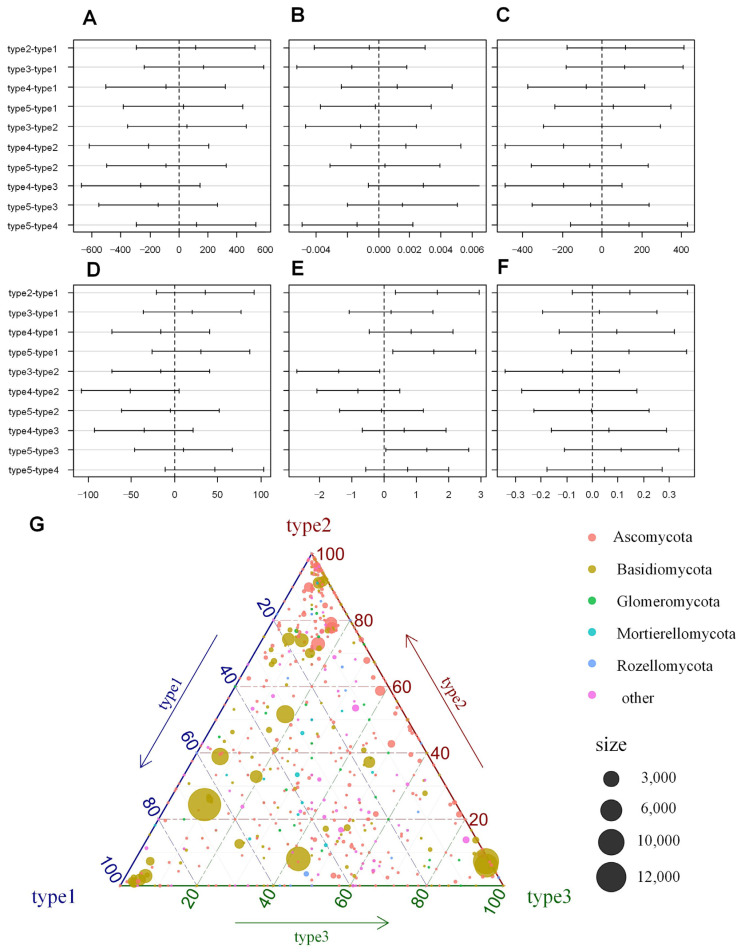
Alpha diversity indices and community composition differences of phyllosphere fungi among different plant types. (**A**–**F**) The results of multiple comparative analysis (Tukey HSD) of alpha diversity indices (Chao1, good’s coverage, observed species, PD whole tree, Shannon, and Simpson) at the 95% family-wise confidence level. (**G**) A ternary plot of phylum-level composition differences among plant types 1, 2, and 3. Point positions indicate the proportional distribution of each phylum across the three plant types, while point sizes represent total sequence reads across all samples. Point sizes were scaled for visualization clarity given the wide range of sequence read counts (e.g., 3000, 6000, 10,000, 12,000).

High-throughput sequencing identified a total of 2901 OTUs across all phyllosphere types. The sequencing coverage for all five types exceeded 99% suggested that the sequencing depth was adequate to accurately capture the fungal community structure. Alpha diversity analysis revealed contrasting patterns among different diversity metrics. Richness-based indices including Chao1, observed species count, and phylogenetic diversity showed no significant differences among the five vegetation types (Figure 2A,C,D,F), indicating that different plant types supported similar numbers of fungal species. However, the Shannon diversity index, which incorporates both richness and evenness components, revealed significant differences between specific plant type pairs (type2 vs. type1, type5 vs. type1, type3 vs. type2, and type3 vs. type5, α = 0.05; Figure 2E). These results suggest that while species richness remained relatively constant, community evenness varied substantially across plant types. Detailed alpha diversity metrics and relative abundance data with standard deviations are presented in Table 2. The apparent contradiction between similar alpha diversity (except Shannon index) and distinct community composition can be explained by species turnover patterns. As shown in Table 2, while most vegetation types supported similar numbers of fungal species (Chao1: 712–975; observed species: 439–636), the Shannon diversity differences (3.96–5.59) suggest variations in community evenness rather than richness.

Community composition analysis revealed that Tremellomycetes were consistently abundant across all types, particularly in type1, type3, and type4, indicating compositional dominance. Dothideomycetes and Sordariomycetes were more abundant in type2 and type5, potentially reflecting plant species-specific microenvironmental differences. Eurotiomycetes showed a notable increase in type5, while Microbotryomycetes displayed a decreasing trend from type1 to type5, indicating sensitivity to plant-mediated environmental differences across wetland vegetation types. Although less abundant, fungal classes such as Mortierellomycetes and Glomeromycetes were consistently detected across all types, implying a stable functional presence in the phyllosphere ecosystem.

The ternary plot illustrating the relative abundances of fungal phyla across type1, type2, and type3 revealed clear distributional patterns among major taxonomic groups. Ascomycota, Basidiomycota, Glomeromycota, Mortierellomycota, Rozellomycota, and other unclassified phyla showed distinct spatial tendencies within the compositional space. Among them, Basidiomycota exhibited a broad distribution across the ternary space, suggesting their adaptability to varying plant-associated environmental conditions. In contrast, Ascomycota and Mortierellomycota displayed more localized clustering, suggesting a stronger preference or specialization along particular environmental gradients. Point sizes, representing fungal abundance, varied substantially, with larger values (e.g., 3000, 6000, 10,000, 12,000) concentrated near specific vertices of the plot, thereby reflecting the dominance of certain phyla under particular conditions (Figure 2G).

### 3.2. Fungal Community Assembly Processes

**Figure 3:**
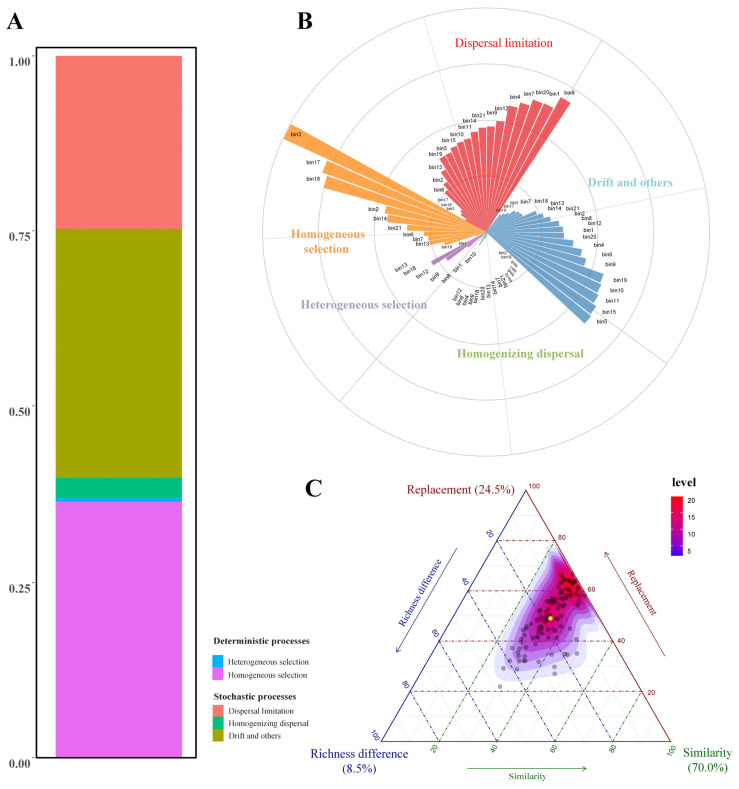
Integrated analysis of Beta diversity components and ecological assembly processes in the phyllosphere fungal community. (**A**) The overall contributions of deterministic processes (e.g., heterogeneous selection, homogeneous selection) and stochastic processes (e.g., dispersal limitation, homogenizing dispersal, and undominated processes) to community assembly. (**B**) The process contributions within each taxonomic bin of the community. (**C**) The ternary plots of Beta diversity components-turnover, nestedness, and richness difference—illustrating how different pairwise dissimilarities are dominated by specific components.

Analysis of the phyllosphere fungal community assembly mechanisms revealed that homogeneous selection (HoS) and drift and other processes (DR) were the predominant drivers, accounting for 36.48% ± 2.1% and 35.49% ± 1.8%, respectively (Figure 3A). This indicates that the fungal community structure in the phyllosphere is shaped by both deterministic environmental filtering and stochastic processes. Dispersal limitation (DL) contributed 24.70% ± 2.5%, reflecting the role of spatial isolation or limited dispersal ability in shaping community distribution. In contrast, heterogeneous selection (HeS) and homogenizing dispersal (HD) showed minimal influence, accounting for only 0.51% ± 0.01% and 2.83% ± 0.07%, respectively, suggesting that environmental heterogeneity and high dispersal rates play limited roles in community assembly in this region. Among the 21 bins, DL was the most frequent dominant process, governing 11 bins (52.4%); DR and HoS each dominated 5 bins (23.8%). HeS and HD were not identified as dominant in any bin, reinforcing their limited role in phyllosphere community assembly within this study. The importance values further supported this trend: bins dominated by DL (e.g., bin1, bin8, bin20) had relatively high values (0.471–0.689), highlighting the key role of dispersal constraints. In HoS-dominated bins, bin16 exhibited the highest importance (0.997), suggesting that fungal communities in this bin were strongly shaped by uniform environmental filtering. DR-dominated bins also had high contribution values (e.g., bin5 = 0.601, bin10 = 0.566), indicating the significant role of stochasticity in certain fungal assemblages (Figure 3B). Overall, fungal community assembly across bins exhibited spatial heterogeneity, with neutral processes (dispersal limitation and drift) being predominant, while deterministic processes (especially HoS) were also important in specific bins (Figure 3C).

### 3.3. Phyllosphere Fungi Distribution Patterns

**Figure 4:**
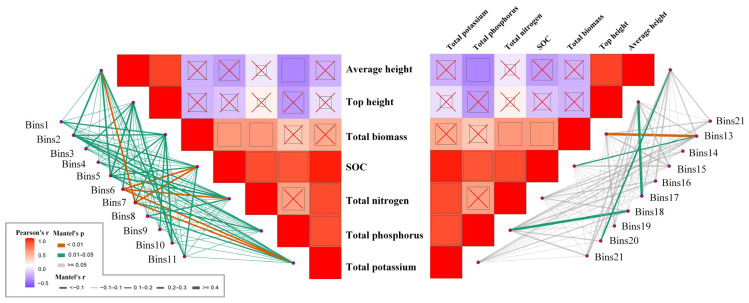
Environmental drivers of phyllosphere community composition. Pairwise comparisons of environmental factors are shown, with a color gradient denoting Spearman’s correlation coefficients and ‘x’ indicating no significant correlation. Community composition of bins was related to each environmental factor by Mantel tests (betaMNTD-corrected). Edge width corresponds to the Mantel’s r statistic and edge color denotes the statistical significance based on 9999 permutations.

Among the 21 fungal community bins analyzed, heterogeneous selection (HeS) played a crucial role in shaping the assembly of bin13 and bin18. Specifically, bin13, in which HeS was identified as a significant contributing mechanism, showed a strong and significant correlation with total biomass, indicating that spatially varying environmental conditions associated with biomass levels may drive niche differentiation in this community. Similarly, bin18 was significantly associated with total phosphorus, suggesting that phosphorus heterogeneity across sampling sites could have contributed to divergent selective pressures influencing community structure. In contrast, homogeneous selection (HoS) dominated bin 2, bin 3, bin 6, and bin 17, reflecting the influence of uniform environmental filtering in shaping these fungal communities. Notably, bin2 was significantly correlated with multiple environmental variables (Figure 4), including average height, soil organic carbon (SOC), total biomass, total nitrogen, and total potassium. This pattern suggests that consistent abiotic conditions across sites exert strong and uniform selective pressures on fungal species composition in this bin. Bin3 was significantly associated with all measured environmental variables, reinforcing the notion that this fungal community is highly structured by broadly homogeneous environmental filters. Bin17 was significantly correlated with top height, implying that uniform canopy structure may be a key determinant shaping fungal composition through consistent light, humidity, or temperature conditions across the sampled area.

### 3.4. Network Structure and Topological Roles of Phyllosphere Fungal Communities

**Figure 5:**
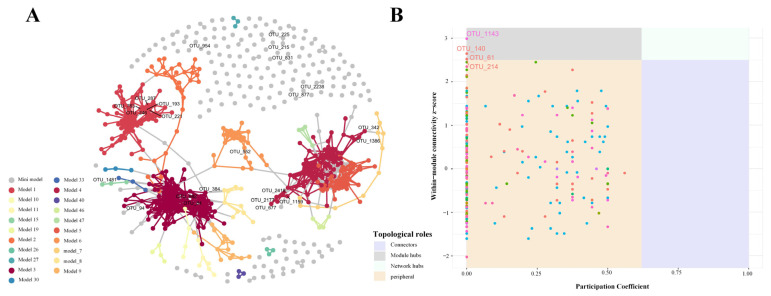
Co-occurrence network and Zi-Pi plot of phyllosphere fungal communities. (**A**) Each node represents an OTU and edges indicate correlations between them. Node colors correspond to different modules. (**B**) Each dot represents an OTU plotted according to its within-module connectivity (Zi) and participation coefficient (Pi).

Co-occurrence network analysis revealed that phyllosphere fungal communities exhibited a highly modular structure. The network was divided into multiple modules, with major modules (Modules 1, 2, and 4, Figure 5A) containing clusters of densely connected OTUs, while numerous peripheral OTUs were also present. Topological role analysis identified four module hubs with high within-module connectivity (Zi > 2.5): OTU_1143 (Bin3), OTU_140 (Bin11), OTU_61 (Bin9), and OTU_214 (Bin7). These hub OTUs were distributed across different ecological bins representing distinct community assembly processes. Environmental variable correlation analysis showed that OTU_1143 had significant associations with SOC, total nitrogen, potassium, biomass, and plant height. OTU_61 showed moderate correlation with soil potassium, while OTU_214 exhibited correlations with total nitrogen and biomass (Figure 4).

## 4. Discussion

### 4.1. Plant Type Drives Leaf Surface Fungal Community Diversity and Differentiation

In this study, a total of 2901 OTUs were obtained, with sequencing coverage exceeding 99%, indicating that the sequencing depth was adequate to accurately represent the structure of the leaf surface fungal community. Alpha diversity analysis showed differential responses among indices, with Shannon diversity varying significantly between plant types while richness indices (Chao1, observed species, phylogenetic diversity) remained constant. This pattern indicates that plant species influence community structure through species turnover rather than changes in overall species richness. This finding is consistent with previous studies, such as a comparison of 6 families and 12 species of plants, where the diversity of leaf surface fungal communities was primarily explained by the host species [46]. This supports plant-mediated species turnover in phyllosphere fungal communities. Further community composition analysis revealed distinct differences in the distribution of fungal classes among different plant types at the class level. Tremellomycetes dominated across all types, particularly in type1, type3, and type4, where their relative abundance was the highest. The high abundance of Tremellomycetes in these types is closely related to the class’s strong adaptation to leaf surface environments [47]. Meanwhile, Dothideomycetes and Sordariomycetes were enriched in type2 and type5, likely reflecting their selective response to the specific chemical environments of particular plant species. This suggests that plant-specific factors contribute to fungal community differentiation, though the underlying mechanisms require further investigation.

The ternary plot analysis further revealed the differing environmental adaptability of fungal phyla. Basidiomycota exhibited a broader distribution across different plant types, indicating their stronger environmental adaptability, while Ascomycota and Mortierellomycota were more concentrated, suggesting these groups may have more specialized environmental requirements. These patterns indicate that plant identity serves as an important environmental filter for phyllosphere fungal communities, though the specific plant traits responsible for this filtering effect remain to be determined. The observed plant-type specific fungal community patterns may result from various plant-associated factors that create unique microhabitats for fungal colonization. Previous studies on plant–microbe interactions have identified potential mechanisms that may be relevant to phyllosphere communities. For example, research on grass–fungal interactions showed recruitment of antagonistic fungi through jasmonic acid pathways [26], while studies on crop plants demonstrated enrichment of specific fungal taxa in disease-resistant varieties [27]. Although these studies were conducted in different plant systems and environments than our constructed wetland setting, they provide insights into possible plant-mediated mechanisms that could influence fungal community assembly.

### 4.2. Multiple Driving Mechanisms of Leaf Surface Fungal Community Assembly

The results from the iCAMP model analysis in this study indicate that homogeneous selection (HoS, 36.48%) and random processes (drift and others, DR, 35.49%) are the main mechanisms driving the assembly of the leaf surface fungal community, followed by dispersal limitation (DL, 24.70%). In contrast, heterogeneous selection (HeS, 0.51%) and homogenizing dispersal (HD, 2.83%) have very limited contributions. This pattern reflects the unique ecological dynamics of the leaf surface environment in community assembly. At a finer bin level, dispersal limitation dominated 11 bins (52.4%), while drift and other processes and homogeneous selection each dominated 5 bins (23.8% each), with HeS and HD not dominating any bins. The importance values of DL-dominated bins were relatively high (0.471–0.689), indicating a stable effect on multiple fungal groups. HoS-dominated bin16 had a particularly high importance value of 0.997, reflecting strong selective pressure. DR-dominated bins such as bin5 (0.601) and bin10 (0.566) also showed significant contributions to community assembly.

These patterns reveal underlying ecological drivers shaping the phyllosphere fungal community. First, the dominant role of homogeneous selection reflects the relatively uniform environmental conditions of leaf surfaces—limited nutrient availability, minimal pH variation, and stable microclimate—which create consistent ecological filters. This is in agreement with previous studies showing that leaf surfaces and other stable microhabitats often exhibit strong homogeneous selection [17,48]. Second, the high contribution of random processes (drift and others) indicates that stochastic events play a major role. The leaf surface is an open system directly exposed to the atmosphere, making fungal colonization highly dependent on chance spore deposition influenced by airflow direction, humidity, and rainfall frequency. In addition, the small physical size of leaves and limited community sizes amplify ecological drift, a pattern observed in other early-stage or disturbance-prone ecosystems [49,50]. Lastly, the importance of dispersal limitation (DL) may reflect the spatial structure and ecological stage of the studied system. As a newly established ecosystem (only three years since planting), the artificial wetland features noticeable spatial isolation between different plant individuals, and the leaf surface microhabitats are relatively independent. The physical pathways for fungal migration between plants are limited. These characteristics lead to localized distributions of certain fungal groups on specific plants, thereby enhancing the effects of dispersal limitation [51]. Similar patterns have been observed in other regions: in northeastern Germany, environmental filtering was identified as a strong driver of phyllosphere fungal differences across three sites [52], whereas a study across six geographically distinct sites in southeastern China found that stochastic processes were the key factors shaping fungal community composition [53]. Together, these comparisons suggest that both deterministic and stochastic forces influence phyllosphere fungal assembly, but their relative importance may vary depending on regional conditions, vegetation characteristics, and successional stages.

### 4.3. Ecological Implications of Network Structure and Topological Roles in Phyllosphere Fungal Communities

The highly modular structure of phyllosphere fungal networks reflects the existence of functionally related taxa that share similar ecological niches and environmental preferences [54,55,56]. The Zi-Pi plot analysis provides a robust framework for characterizing topological roles: the within-module degree (Zi) quantifies the connectivity of a taxon within its own module, while the participation coefficient (Pi) measures the extent to which its connections are distributed across different modules [55]. This dual-metric approach facilitates the identification of keystone taxa with disproportionate influence on community structure [55]. The modular organization, characterized by dense within-module connections and sparse between-module connections, suggests functional differentiation representing different trophic strategies such as saprophytic decomposers or endophytic symbionts [57,58]. The numerous peripheral OTUs, despite low connectivity, may serve as reservoirs of functional diversity and contribute to ecosystem stability during environmental perturbations [59].

Four module hubs with high within-module connectivity (Zi > 2.5) were identified: OTU_1143 (Bin3), OTU_140 (Bin11), OTU_61 (Bin9), and OTU_214 (Bin7). The lack of network hubs (Zi > 2.5, Pi > 0.62) suggests functional compartmentalization rather than universal connectivity [60]. OTU_1143, under strong homogeneous selection, exhibited extensive environmental associations and represents a generalist keystone species maintaining community stability [61]. OTU_140 emerged as a stochastic hub shaped by historical contingency and priority effects rather than environmental filtering [62]. OTU_61 functions as a specialized hub sensitive to environmental gradients, potentially serving as an early warning indicator of ecosystem change [63,64]. OTU_214 represents a context-dependent hub balancing environmental selection and biotic interactions [65]. These hub taxa demonstrate how different assembly mechanisms (deterministic vs. stochastic) can generate functionally important network positions.

The integration of environmental factors and network topology reveals multi-level regulatory mechanisms where environmental filtering initially selects adaptive taxa, which then form interaction networks based on niche complementarity and resource competition [66]. Environmental variables not only directly affect taxon abundance but also indirectly shape network structure by modulating interspecies interactions [23]. Plant morphological traits create microenvironmental gradients that promote niche differentiation and influence co-occurrence patterns [67]. Understanding these environment–network–function coupling mechanisms provides crucial insights for constructed wetland management [17]. The identification of hub taxa and their environmental drivers offers targets for bioaugmentation strategies to enhance pollutant removal and nutrient cycling efficiency, while the modular organization suggests that management interventions should consider functional groups to avoid system-wide disruptions [68].

### 4.4. Study Limitations

While our results support plant-mediated structuring of phyllosphere fungal communities, several limitations deserve consideration. Methodological limitations include the examination of a limited number of plant types (*n* = 3) at a single geographic location, which may constrain the applicability of our findings to broader wetland ecosystems [69]. Our single-timepoint sampling during the growing season failed to capture seasonal variations in plant physiology, environmental conditions, and fungal community dynamics that could significantly influence community patterns. Additionally, microclimate variables such as local humidity, temperature fluctuations, and light exposure were not measured, yet these factors may contribute to the observed patterns. The ITS1-based approach, while standard for fungal community analysis, may not capture the complete taxonomic diversity, particularly for groups with poor amplification efficiency.

## 5. Conclusions

This study revealed that plant type significantly shaped foliar fungal communities in the constructed wetland, with Tremellomycetes being the dominant class. Their high relative abundance may be related to adaptation to the phyllosphere environment, but this requires further experimental validation. Community assembly was jointly driven by heterogeneous selection (36.48%), dispersal-related processes (35.49%), and drift (24.70%), indicating that both deterministic and stochastic processes play comparable roles. Co-occurrence network analysis further revealed modular structures with key hub taxa that may contribute to network stability. Together, diversity analysis, iCAMP-based assembly inference, and network analysis provide a complementary and integrated understanding of fungal community structure and potential ecological functions. These findings offer potential guidance for wetland restoration and vegetation management by suggesting that plant type selection can influence microbial communities, and hub taxa could be considered as potential targets for bioaugmentation to enhance ecosystem stability. However, our results are based on a single restored wetland converted from farmland, and conclusions may not be generalizable to other wetland types or regions. Future studies should include multi-site and multi-season sampling, incorporate plant functional traits and soil properties, and test the functional roles of hub taxa experimentally to validate and expand the applicability of these findings.

## Figures and Tables

**Table 1:** Types of constructed wetlands with plant composition.

Type ID	Plant Composition	Cover Contribution (%)
1	*Canna indica* L.	90
2	*Cyperus alternifolius* L.	95
3	*Typha orientalis* Presl	95
4	*Thalia dealbata* Fraser	30
	*Canna indica*	30
	*Typha orientalis*	30
5	*Cyperus alternifolius*	50
	*Typha orientalis*	40

**Table 2:** Alpha diversity indices and relative abundance of phyllosphere fungal communities across different constructed wetland vegetation types.

Type ID	Chao1	Good’s Coverage	Observed Species	PD Whole Tree	Shannon	Simpson	Relative Abundance
1	802.03 ± 140.01	0.99 ± 0.00	518.97 ± 107.98	119.06 ± 18.42	3.96 ± 0.58	0.80 ± 0.14	0.06 ± 0.09
2	919.40 ± 23.33	0.99 ± 0.00	635.67 ± 29.11	155.03 ± 22.29	5.59 ± 0.38	0.94 ± 0.02	0.09 ± 0.13
3	975.15 ± 128.98	0.99 ± 0.00	632.90 ± 94.07	139.18 ± 13.48	4.17 ± 0.39	0.83 ± 0.04	0.07 ± 0.11
4	712.06 ± 35.96	0.99 ± 0.00	439.33 ± 10.44	103.75 ± 7.56	4.79 ± 0.08	0.89 ± 0.01	0.08 ± 0.11
5	832.98 ± 154.24	0.99 ± 0.00	573.00 ± 99.33	150.11 ± 10.12	5.50 ± 0.08	0.94 ± 0.00	0.04 ± 0.07

## Data Availability

The raw data supporting the conclusions of this article will be made available by the authors on request.
